# The expression and localization of V-ATPase and cytokeratin 5 during postnatal development of the pig epididymis

**DOI:** 10.5713/ajas.19.0587

**Published:** 2019-11-12

**Authors:** Yun-Jae Park, Ji-Hyuk Kim, Hack-Youn Kim, Hee-Bok Park, Juhui Choe, Gye-Woong Kim, Sun-Young Baek, Hak-Jae Chung, Yoo-Jin Park, Bongki Kim

**Affiliations:** 1 Department of Animal Resources Science, Kongju National University, Yesan 32439, Korea; 2 Division of Swine Science, National Institute of Animal Science, Rural Development Administration, Cheonan 31172, Korea; 3 Department of Animal Science and Technology, Chung-Ang University, Anseong 17546, Korea

**Keywords:** Clear Cell, Basal Cell, Immunofluorescence, Epithelium, Luminal Environment

## Abstract

**Objective:**

We examined the localization and expression of H^+^ pumping vacuolar ATPase (V-ATPase) and cytokeratin 5 (KRT5) in the epididymis of pigs, expressed in clear and basal cells, respectively, during postnatal development.

**Methods:**

Epididymides were obtained from pigs at 1, 7, 21, 60, 120, and 180 days of age; we observed the localization and expression patterns of V-ATPase and KRT5 in the different regions of these organs, namely, the caput, corpus, and cauda. The differentiation of epididymal epithelial cells was determined by immunofluorescence labeling using cell-type-specific markers and observed using confocal microscopy.

**Results:**

At postnatal day 5 (PND5), the localization of clear cells commenced migration from the cauda toward the caput. Although at PND120, goblet-shaped clear cells were detected along the entire length of the epididymis, those labeled for V-ATPase had disappeared from the corpus to cauda and were maintained only in the caput epididymis in adult pigs. In contrast, whereas basal cells labeled for KRT5 were only present in the vas deferens at birth, they were detected in all regions of the epididymis at PND60. These cells were localized at the base of the epithelium; however, no basal cells characterized by luminally extending cell projections were observed in any of the adult epididymides examined.

**Conclusion:**

The differentiation of clear and basal cells progressively initiates in a retrograde manner from the cauda to the caput epididymis. The cell-type-specific distribution and localization of the epithelial cells play important roles in establishing a unique luminal environment for sperm maturation and storage in the pig epididymis.

## INTRODUCTION

The epididymis is a highly convoluted duct that connects the testis and vas deferens and is anatomically divided into three regions, the caput, corpus, and cauda. In these regions, the epididymal lumen is lined by a pseudo-stratified epithelium composed of three major cell types, namely, the principal (PCs), clear (CCs), and basal (BCs) cells that interact cooperatively to establish an optimal luminal environment for the maturation and storage of spermatozoa [1,2]. PCs, the most abundant cell type in the epididymis, synthesize and secrete proteins, zona pellucida binding protein dicarbonyl/1-xylulose reductase (P26H/P34H/DCXR) [3], macrophage migration inhibitory factor [4], and epididymal secretory protein E5 (HE5/CD52) [5], and also reabsorb and secrete bicarbonate via the cystic fibrosis transmembrane regulator [6]. These cells play essential roles in promoting functional maturity and establishing a unique storage environment for spermatozoa. CCs in all regions of the epididymis secrete protons via the H^+^ pumping vacuolar ATPase (V-ATPase) located in the apical membrane of these cells [7]. V-ATPase plays an important role in establishing a luminal acidic pH in several organs, including the epididymis, kidney, lung, and inner ear [7–10]. In addition, CCs are endocytic cells that remove proteins from the epididymal fluid and cytoplasmic droplets from sperm during epididymal transit [11]. BCs located adjacent to epithelial cells have been shown to have self-renewal properties and the capacity to differentiate into several epithelial cell types in the trachea and prostate [12,13]. A number of studies have reported that BCs are restricted to the basal region of pseudo-stratified epithelia. Recently, however, it has been reported that BCs have highly plastic morphological characteristics in the epididymis. For example, in mice and rats, the cytoplasmic cell bodies of BCs can extend, pass through tight junctions, and come into contact with the epididymal lumen [14,15]. In addition, Park et al [16] have reported that crosstalk and collaboration among these epithelial cells are necessary for maintaining the unique luminal environment. Accordingly, determining the development and differentiation of epithelial cells in the epididymis is essential for gaining an understanding of the mechanisms underlying the establishment of the unique environment of the epididymal lumen.

In rats, the postnatal development of the epididymis can be divided into three phases, namely, the undifferentiated period, the differentiation period, and a subsequent period of expansion [17], and these phases of postnatal development have been examined using specific markers for PCs (AQP9) and CCs (V-ATPase) [18,19]. In contrast, very little is known regarding the development and differentiation of epididymal epithelial cells during postnatal development in pigs. Furthermore, the mechanisms that contribute to establishing the optimal luminal environment for sperm maturation and storage via luminal acidification have yet to be determined in pigs. In the present study, we accordingly sought to examine the expression and localization of V-ATPase and cytokeratin 5 (KRT5) to respectively characterize the differentiation of CCs and BCs in the pig epididymis.

## MATERIALS AND METHODS

### Animals

Epididymides and vas deferens were collected from the pre-pubertal (tissues collected at 1-, 7-, 21-, and 60 days of age), pubertal (tissues collected at 120 days of age), and post-pubertal (tissues collected at 180 days of age) stages in Landrace × Large White Yorkshire cross-bred pigs [20]. There was no spermatozoa found in the epididymis until 120 days of age, and the spermatozoa was only observed in the epididymis at 180 days of age. All procedures described were reviewed and approved by the Institutional Animal Care and Use committee at the National Institute of Animal Science (Approval No. NIAS2019-117).

### Tissue fixation and preparation

Following harvest, the epididymides and vas deferens were fixed by immersion in 4% paraformaldehyde dissolved in phosphate-buffered saline (PBS) for 24 h at room temperature and thereafter given five 20-min washes in PBS. Tissues were then incubated in a solution of 30% sucrose in PBS for at least 24 h. The tissues were subsequently embedded in OCT compound (Tissue-Tek; Sakura Finetek, Torrance, CA, USA) mounted on a cutting block, and frozen. The tissues were cut using a Leica 3050S cryostat (Leica Microsystems, Bannockburn, IL, USA) at thickness of 10 μm for normal staining and 16 μm for 3D reconstructions, placed onto Fisher Superfrost/Plus microscope slides (Fisher Scientific, Pittsburgh, PA, USA), and stored at −20°C until use.

### Immunofluorescence staining and antibodies

The cryo-sections were hydrated in PBS for 10 min, and heated by microwave in a 10 mM Tris/1 mM ethylenediaminetetraacetic acid buffer (pH 9.0) for antigen retrieval (three 2-min treatments with a 5-min interval between treatments). Non-specific binding was blocked by incubation with 1% bovine serum albumin in PBS for 60 min at room temperature. The sections were then incubated with primary antibodies in a moist chamber for 90 min at room temperature or overnight at 4°C. Thereafter, the samples were washed in PBS and incubated with secondary antibodies for 60 min at room temperature. The slides were washed and then placed in Vectashield medium (Vecta Labs, Burlingame, CA, USA) containing 4′, 6-diamidino-2-phenylindole to label the nuclei. For CC identification, an affinity-purified chicken polyclonal antibody against the B1 subunit of V-ATPase (B1-V-ATPase) (diluted 1:800) was used as described previously [1]. For BC identification, a rabbit monoclonal antibody against the C terminus of KRT5 (Thermo Scientific, Rockford, IL, USA) (diluted 1:300) was applied [21]. We also used a mouse monoclonal anti-ZO1 antibody (Thermo Scientific, USA) (diluted 1:200) [22] to identify the BC projections that reached to the epididymal lumen. The following secondary antibodies were obtained from the Jackson ImmunoResearch Laboratories (West Grove, PA, USA): Cy3-conjugated donkey anti-chicken immunoglobulin G (IgG); fluorescein isothiocyanate (FITC)-conjugated donkey anti-rabbit IgG; and FITC-conjugated donkey anti-mouse IgG. For a negative control, slides were incubated with rabbit or mouse immunoglobulin fraction (DAKO, Carpinteria, CA, USA) instead of the primary antibody. All antibodies were diluted in Dako antibody diluent (DAKO, USA). Confocal images were acquired using a Zeiss confocal microscope (LSM800) and analyzed using Zen Blue software. The final images were generated using Adobe Photoshop software.

### Quantification of clear and basal cells

The CCs and BCs were determined as the number of cells that were positive for B1-VAPTase and KRT5, respectively. At least three epididymides were examined at each of the designated age points. These numbers were normalized according to the total number of BCs and CCs present in each tubule section examined. Digital images were acquired with a ×20 objective using a Zeiss confocal microscope and were analyzed using Zen Blue software.

### Statistical analysis

For each of the sampling time points, we examined the epididymides from at least three pigs and for each epididymis we analyzed three cryostat sections. Comparisons between multiple groups were performed using a general linear model followed by Tukey simultaneous tests. The threshold of statistical significance was set at p = 0.02.

## RESULTS

### Expression of B1-VAPTase and KRT5 in the pig epididymis

**Figure 1:**
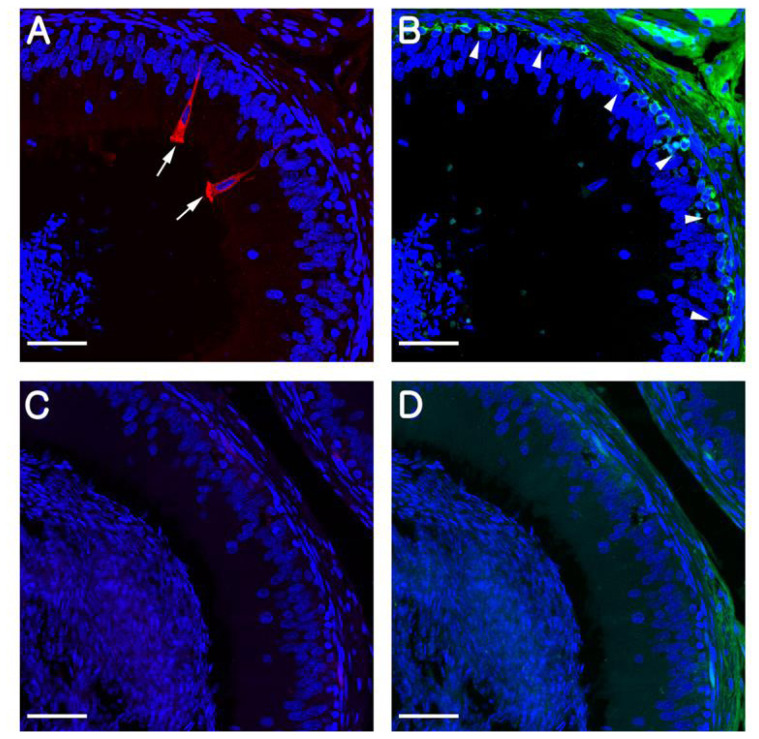
Immunolocalization of B1-VATPase (red) and KRT5 (green) in the epididymis at postnatal day (PND) 180. (A) A pig epididymis section labeled with anti-B1-VATPase. Goblet-shaped clear cells were observed (arrows). (B) A pig epididymis section labeled with anti-KRT5. Basal cells were located at the base of the epithelium (arrowheads). (C and D) Negative controls showed no B1-VAPTase or KRT5 staining in the epididymis. Nuclei are labeled with DAPI (blue). V-ATPase, vacuolar ATPase; KRT5, cytokeratin 5; DAPI, 4′, 6-diamidino-2-phenylindole. Bars = 50 μm.

In rodents, B1-VAPTase and KRT5 are typically used as markers for CCs and BCs, respectively, and in the present study, we confirmed that these markers also specifically label CCs and BCs in the pig epididymis. Consistent with previous observations in mice and rats, we observed that B1-VATase is exclusively expressed in the cytoplasm of the CCs, and that KRT5 is clearly expressed in the cytoplasm of BCs located at the base of the epithelium (Figure 1A, B). No labeling was detected when the B1-VATPase and KRT5 primary antibodies were omitted from tissue preparations (Figure 1C, D).

### Differentiation of clear cells during postnatal development

**Figure 2:**
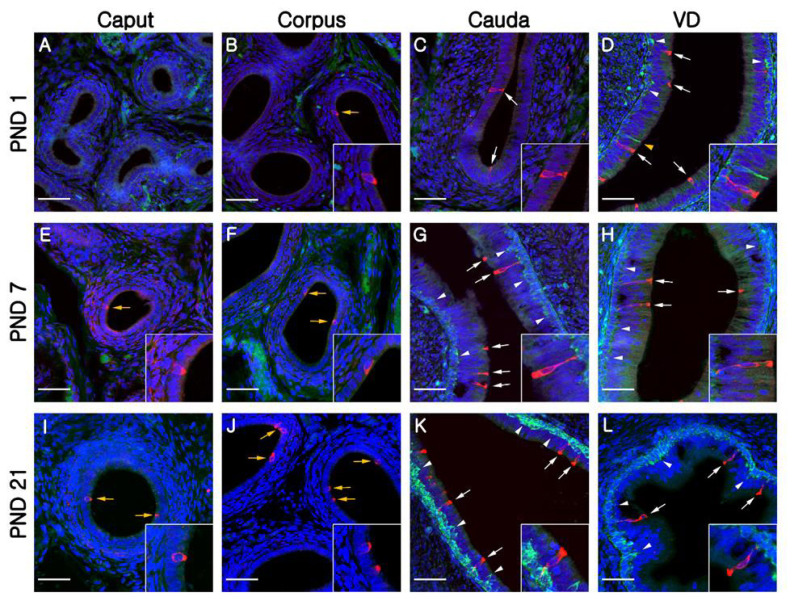
Progressive appearance of clear cells and basal cells from the VD to the caput of epididymides during the first three postnatal weeks. Cells in the epididymis were double-labeled for B1-VATPase (red) and KRT5 (green). (A–D) Epididymis at PND1. (E–H) Epididymis at PND7. (I–L) Epididymis at PND21. Yellow and white arrows indicate round- or goblet-shaped clear cells, respectively. Yellow and white arrowheads indicate projecting or dome-shaped basal cells, respectively. Nuclei are labeled with DAPI (blue). VD, vas deferens; V-ATPase, vacuolar ATPase; KRT5, cytokeratin 5; PND, postnatal day; DAPI, 4′, 6-diamidino-2-phenylindole. Bars = 50 μm.

**Figure 3:**
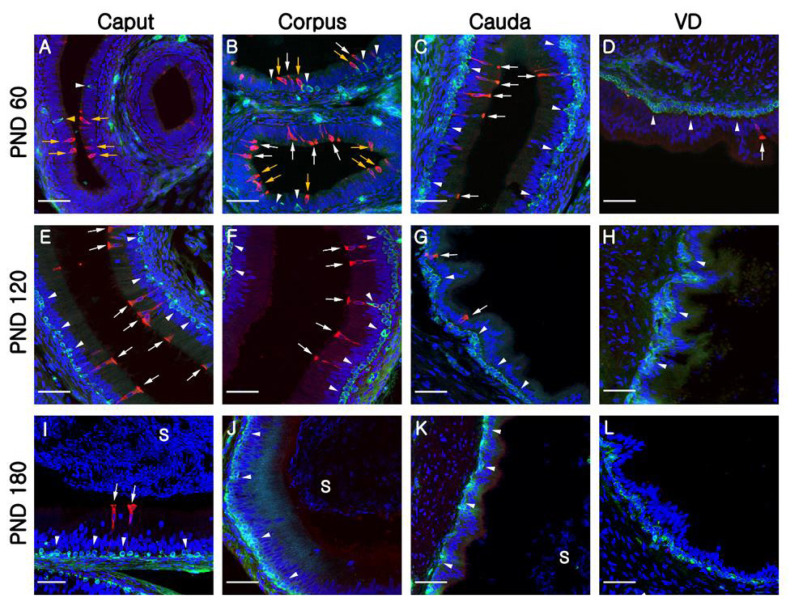
Epididymis from PND60 to PND180. Cells in the epididymis were double-labeled B1-VATPase (red) and KRT5 (green). (A–D) Epididymis at PND60. (E–H) Epididymis at PND120. No labeled clear cells were observed in the VD at PND120. (I–L) Epididymis at PND180. Clear cells are only labeled in the caput epididymis. Yellow and white arrows indicate round- or goblet-shaped clear cells, respectively. Yellow and white arrowheads indicate projecting or dome-shaped basal cells, respectively. S, spermatozoa. Nuclei are labeled with DAPI (blue). PND, postnatal day; V-ATPase, vacuolar ATPase; KRT5, cytokeratin 5; VD, vas deferens; DAPI, 4′, 6-diamidino-2-phenylindole. Bars = 50 μm.

**Figure 4:**
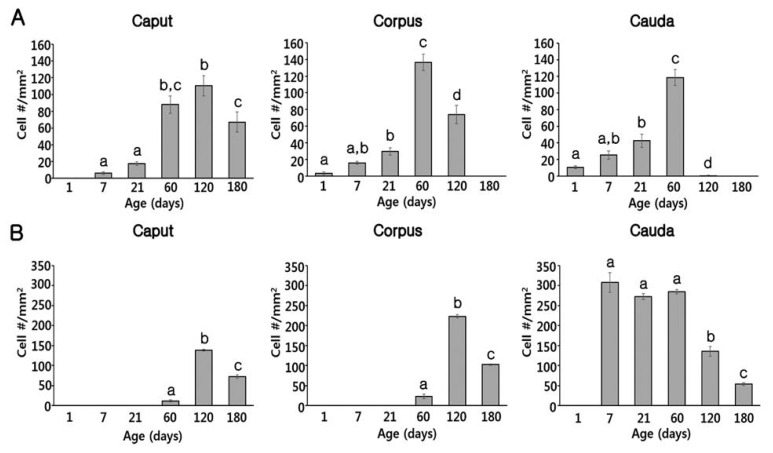
Quantitative changes in cell numbers during postnatal development of the pig epididymis. (A) Changes in the number of clear cells in the caput, corpus, and cauda. (B) Change in the volume of basal cells in the caput, corpus, and cauda. Cell numbers were calculated based on the number of clear and basal cells in the epithelium per square millimeter of the epididymal area. Results are expressed as means±standard error of the mean. Means with different letters differ (p<0.05).

At PND1, B1-VATPase-labeled CCs were observed from the proximal vas deferens to the cauda and corpus regions of the epididymis (Figure 2B–2D), although they were not detected in the caput (Figure 2A). Within the vas deferens, we observed numerous CCs characterized by a goblet-shaped morphology (Figure 2D, white arrows). In the epididymis, however, CCs with a round shape or goblet-like appearance were only occasionally detected in the corpus and cauda, respectively (Figure 2B, yellow arrow; Figure 2C, white arrows). At PND7 and 21, goblet-shaped CCs were detected in the cauda and vas deferens (Figure 2G, 2H, 2K, and 2L, white arrows), whereas a small number of CCs with a round morphology were detected in the caput and corpus (Figure 2E, 2F, 2I, and 2J, yellow arrows). At PND60, we observed that CCs continued to appear progressively in an ascending pattern from the distal region (cauda) to the proximal region (caput) of the epididymis (Figure 3A–3D). However, the morphology of the CCs tended to vary depending on the regional location. Round- and goblet-shaped CCs were mainly present in the caput (Figure 3A, yellow arrows) and cauda (Figure 3C, white arrows), respectively. Within the corpus, we observed both round- and goblet-shaped CCs (Figure 3B). These results indicate that the differentiation of CCs commences from the distal region (cauda) and proceeds to the proximal region (caput) of the epididymis. At PND120, goblet-shaped CCs were observed in all regions of the epididymis (Figure 3E–3G, white arrows). However, we noted a marked decrease in the number of CCs in the cauda (Figures 3G, 4A) and found that they were no longer detectable in the vas deferens (Figure 3H). At PND180, CCs were observed only in the caput (Figures 3A, 4A), all of which were goblet-shaped, and were absent from the vas deferens to the corpus (Figure 3J–3L). Again, these observations indicate that the differentiation of CCs is initiated in the distal region of the epididymis and subsequently proceeds to the proximal region. No labeling was detected when the B1-VATPase primary antibody was omitted from tissue preparations (Supplementary Figure S1A). The changes in the numbers of CCs were examined according to region and age (Figure 4A). In the caput, the number of CCs peaked at PND120 and then significantly decreased at PND180 (Figure 4A). In the corpus and the cauda, quantification analysis (Figure 4A) showed that the number of CCs peaked at PND60 and then considerably decreased at PND120. We did not observed CCs at PND180 from both the corpus and the cauda regions of the epididymis (Figure 4A).

### Differentiation of basal cells during postnatal development

Although BCs labeled for KRT5 were observed in the vas deferens (Figure 2D) at PND1, we were unable to detect any of these cells in the epididymis (Figure 2A–2C). Most BCs in the vas deferens had a dome-shaped morphology and were located beneath other epithelial cells (Figure 2D, white arrowheads); however, we also detected a few BCs with narrow cytoplasmic body projections (Figure 2D, yellow arrowhead). Thereafter, BCs were detected in the cauda region of the epididymis, although they did not progress to the caput and corpus regions until PND21 (Figure 2E–2L). At PND60, most of the BCs detected were still localized in the cauda region; however, some dome-shaped BCs had started to appear in the epididymal corpus (Figure 3B, white arrowheads) At PND120, we observed a progressive appearance of BCs in an ascending pattern from distal regions (cauda) to the more proximal regions (caput) of the epididymis (Figure 3E–3H). At PND180, spermatozoa were detected from the caput to cauda, and BCs were observed along the entire length of epididymis, lying beneath the base of the epithelium (Figure 3I–3L). No labeling was detected when the KRT5 primary antibody was omitted from tissue preparations (Supplementary Figure S1B). In terms of cell numbers, the highest number of BCs in the cauda was observed at PND7 and thereafter continued to decrease until the final observation made at PND180 (Figure 4B). In both the caput and corpus, peak numbers of BCs were observed at PND120. We also observed that BCs in the epididymal epithelium exhibited a diverse range of morphologies that tended to be age and region dependent. Some BCs contained nuclei located at the same height as those in the adjacent epithelial cells (Figure 3A, yellow arrowhead) and some were dome shaped and located beneath other epithelial cells (Figure 3C, white arrowheads), whereas others contained nuclei located in the middle position between two different types of BCs (Figure 3B, white arrowheads).

**Figure 5:**
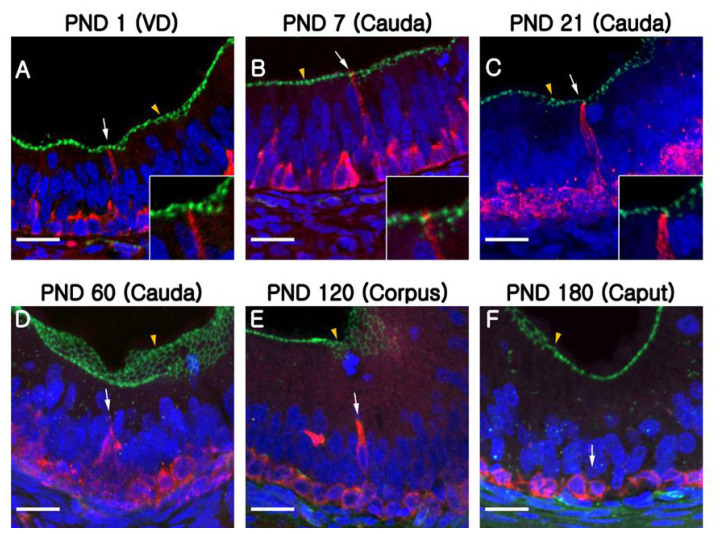
Basal cell projections in the pig epididymis. The basal cells are labeled for KRT5 (red), and tight junctions are labeled for ZO-1 (green). (A–C) During the first 21 days of postnatal development, basal cell projections passed through the tight junctions, thereby coming into contact with the luminal contents (arrows). (D–E) The arrows indicate basal cells with short projections that do not reach the apical border of the epithelium. (F) The arrow indicates basal cells with no projections. The yellow arrowheads indicate tight junctions. Nuclei are labeled with DAPI (blue). KRT5, cytokeratin 5; ZO-1, zonula occludens-1; DAPI, 4′, 6-diamidino-2-phenylindole; PND, postnatal day. Bars = 20 μm.

Quantification analysis of BCs according to region and age is shown in Figure 4B. The BCs peaked at PND120 and were not observed at the caput or the corpus regions of the epididymis until PND21. In the cauda, however, a significantly higher number of BCs was detected from PND7 to 60 compared to those from PND120 and 180, but BCs were absent at PND1 (Figure 4B). Quantification results confirmed that the differentiation of BCs initiated in a retrograde manner from the cauda to the caput region of the epididymis. To determine whether the BCs develop long narrow extensions that project toward the lumen, as seen in the BCs of mice and rats, KRT5 was double labeled with zonula occludens-1 (ZO-1), a tight junction protein (Figure 5). ZO-1 is located in the most apical regions of adhesion between epithelial cells (Figure 5, yellow arrowheads). Although most of the BCs we observed were dome shaped and located at the base of the epithelium, we detected a few BCs with extensions that reached the apical pole of the epithelium during the early stages of postnatal development (PND1 to PND21: Figure 5A–5C). We found that the BCs began to retract from these apical positions at PND60 (Figure 5D, white arrow). We detected no BC projections in the epithelium at PND180, at which time all these cells were observed lying beneath the base of the epithelium (Figure 5F, white arrow).

## DISCUSSION

In this study, we initially examined the early development and differentiation of CCs and BCs in pig epididymis using antibodies directed against B1-VATPase and KRT5, Consistent with the findings of previous studies using rodents, we observed that both of these cell types initially appear in the vas deferens, then in the cauda, and subsequently in more proximal segments of the pig epididymis. Contrastingly, previous studies have indicated that CCs do not appear in the epididymis until PND7 and are present in the caput and cauda of adult epididymides, whereas BCs are detected in all regions of the pig epididymis from PND7 [23]. We suspect that these disparities between observations made in the present and previous studies can be attributed to differences in the techniques used to detect epididymal epithelial cells. In the present study, we used cell-specific markers to identify individual cell types, whereas others have classified cell types based on their morphological properties using hematoxylin and eosin staining. Although we observed differences in the initial development of CCs and BCs, we found that the differentiation of both cell types had reached completion at approximately the same time point of PND120, suggesting the possibility of cell-specific development and differentiation mechanisms. In this regard, we can speculate on the identity of candidate factors that might be implicated in the regulation of cell development and differentiation in the epididymis, including testosterone, testicular luminal factors (TLFs), ROS proto-oncogene 1, receptor tyrosine kinase (ROS1), and dicer1, ribonuclease III (Dicer1). Indeed, recent studies have reported that testosterone plays an important role in epithelial cell proliferation and the expression of AQP9 in PCs [24,25]. It has also been demonstrated that TLFs produced by the testes play important roles in epithelial cell survival and differentiation [21]. In addition, the orphan receptor tyrosine kinase ROS1, which is potentially activated by TLFs, is known as a key player in epithelial cell differentiation. Consistently, it has been demonstrated that male mice in which ROS1 has been knocked out using c-ROS1 oncogene 1 have undifferentiated epithelium [26]. Accordingly, it is probable that multiple factors are involved in the development and differentiation of pig epithelial cells.

### Differentiation of clear cells in the pig epididymis

**Figure 6:**
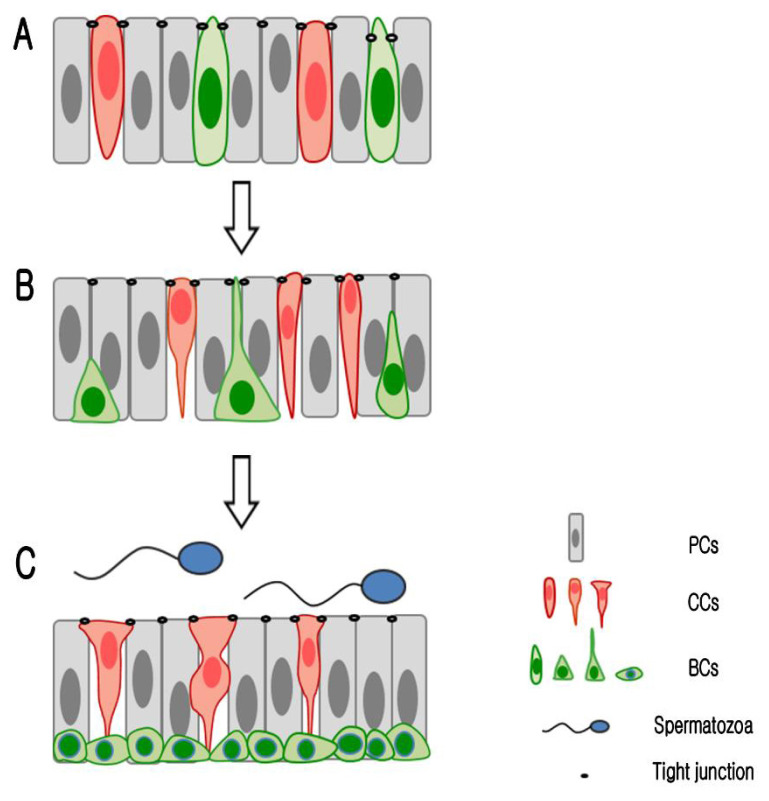
Schematic diagram of epithelial cell differentiation in pig epididymis during postnatal development. (A) During early postnatal development, all epithelial cell types in the pig epididymis have a similar morphological appearance (round- or columnar-shaped). (B) During the pre-puberty stages of development, the different types of epithelial cell undergo morphological differentiation. Whereas principal cells maintain their columnar shape, basal cells occasionally develop narrow or triangular-shaped cytoplasmic cell bodies that can extend to the tight junctions. Moreover, the nuclei of these cells begin to migrate to the base of the epithelium. The morphology of clear cells changes from columnar- to goblet-shaped. (C) In the epididymis of adult pigs, the different types of epithelial cell are morphologically well differentiation. Principal cells have a columnar-shaped cell body, basal cells have a dome-shaped cell body, and clear cells have a goblet-shaped cell body.

As shown in the Figure 6, round- or oval-shaped CCs were observed along the entire length of epididymal duct during epithelial development (Figure 6A), whereas only goblet-shaped CCs were maintained in the caput epididymis after the completion of differentiation (Figure 6C). This is similar to the pattern observed in the vampire bat epididymis, in which only goblet-shaped CCs are observed throughout the epididymis [22]. In mice, however, CCs exhibiting a diverse range of morphologies, including pencil-, cuboidal-, and goblet-shaped cells, are known to co-exist [27,28]. It has been proposed that the morphological development of CCs is a species-specific trait. In this regard, the secretion of H+, promoted by high levels of proton-pumping V-ATPase in the apical membrane of CCs, and the absorption of HCO_3_^−^ by PCs, are necessary for maintaining optimal conditions for sperm maturation and storage in a number of species [16]. In pigs, the cauda epididymis is characterized by a relatively low pH level (6.5) that is significantly lower than that of caput epididymis (pH 7.2) [29]. Interestingly, in the present study, we found that CCs are present in the caput epididymis of adult pigs but not in the corpus and cauda epididymis. Moreover, in some species, including monkeys and bulls, it has been observed that the epididymis is completely devoid of CCs [30,31]. It has been suggested that species-specific mechanisms are required for the maintenance of luminal acidification. Recently, it has been demonstrated that AQP9 is expressed in the nuclei of epithelial cells in the caput and cauda regions of the pig epididymis, whereas in the distal region of the duct, the cauda epididymis, this protein is expressed at the apical surface of the epithelium [23]. These observations suggest the possibility that PCs in the cauda epididymis have an enhanced capacity to absorb HCO_3_^−^, and thus interact to a greater extent with the BCs to regulate luminal acidification [16]. Accordingly, a relatively lower luminal pH can be maintained in the distal cauda region of the pig epididymis compared with the proximal caput region. Further studies are thus warranted to gain a better understanding of the mechanisms underlying regulation of luminal acidification in the pig epididymis.

### Differentiation of basal cells in the pig epididymis

In adult pigs, BCs can readily be distinguished by their location at the base of the epididymis. In contrast, however, as highlighted in the present study, the identification of BCs in the epididymis during early postnatal development is generally considerably more difficult, owing to the co-location of multiple cell types, including PCs, CCs, and BCs. Accordingly, it is necessary to employ cell-specific markers to identify each type of epithelial cell during early postnatal development. Recently, Schimming et al [23] reported that BCs can be detected in all regions of the pig epididymis at PND7. However, in the present study, we were unable to detect the ubiquitous distribution of these cells in the epididymis until PND60. We suspect that these disparate observations could be attributed to the different techniques used to detect BCs. Whereas, Schimming et al [23] used hematoxylin and eosin staining, which can be used to identify the location of BCs based on the staining of their nuclei, we employed immunofluorescence staining using a KRT5 antibody that has been used previously to specifically label BCs in a range of species, including mice, rats, and bats [2,21,22]. In addition, we demonstrate herein that the morphology and localization of BCs undergo significant changes during the course of postnatal development. As illustrated in Figure 6, initially, during early development, BCs are characterized by a round morphology and in these cells the nuclei are located at the same height as those of adjacent epithelial cells (Figure 6A). At a later stage of development, these cells have a narrower or triangular-shaped morphology and their nuclei migrate to the base of the epithelium (Figure 6B). On completion of development, BCs have a hemispherical shape and their nuclei are located at the base of the epithelium (Figure 6C). These observations are consistent with those made in previous rodent studies, which have demonstrated that BCs have considerable structural plasticity during differentiation in rats [2] and mice (unpublished data). However, in the present study, we were unable to detect any narrow cell projections extending toward the lumen, as seen in rats and mice. Previous studies have also reported that the development of BC extensions in adult mice and rats is regulated by TLFs and androgen, respectively [2,21]. Further studies are thus needed to identify the factors regulating the luminally directed development of BC extensions and why such projections are not observed in the BCs of pigs.

In summary, the results obtained in this study reveal that both CCs and BCs progressively initiate in a retrograde manner from the vas deferens and cauda epididymis to the caput. During the course of development, CCs disappear from the region extending from the corpus to cauda, and in adult pigs these cells are present exclusively in the caput epididymis. In contrast to CCs, BCs show significant structural plasticity during postnatal development. Collectively, these findings indicate that appropriate development and differentiation of the epithelium during postnatal stages are necessary to facilitate optimal sperm maturation and storage in the epididymis of pigs.
