# Supplementary material for: The expression and localization of V-ATPase and cytokeratin 5 during postnatal development of the pig epididymis
Source: Asian-Australas J Anim Sci. 2019 Nov 12;33(7):1077–86. doi: 10.5713/ajas.19.0587 (PMC7322665; doi:10.5713/ajas.19.0587)
Supplement: Supplementary file 1 [file ajas-19-0587-suppl1.pdf]

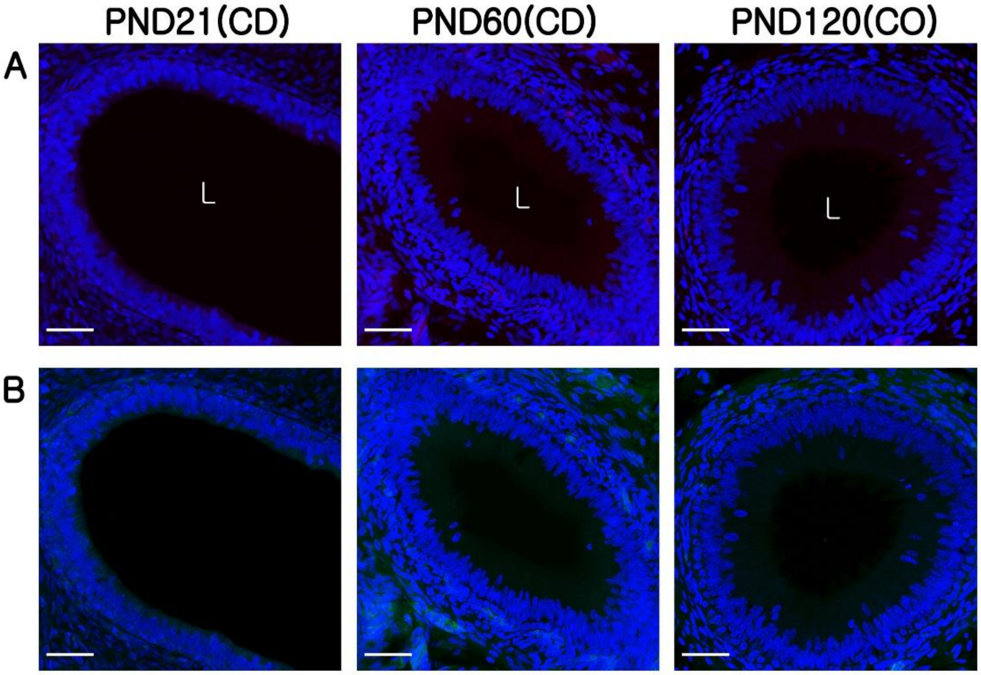

Supplementary Figure S1. Representative images show negative control of immunofluorescence staining in the epididymis. (A) Negative control for B1-VATPase at PND21, 60, and 120. (B) Negative control for KRT5 at PND21, 60, and 120. CO, corpus; CD, cauda; L, lumen. Nuclei are labeled with DAPI (blue). Bars = 20 μm. PND, postnatal day.
